# Low-abundance mutations in colorectal cancer patients and healthy adults

**DOI:** 10.18632/aging.102657

**Published:** 2020-01-12

**Authors:** Yanfei Li, Zhengsheng Dai, Gang Huang, Yueling Jin, Zhongping Ning, Junwei Shen

**Affiliations:** 1 Shanghai University of Medicine and Health Sciences Affiliated Zhoupu Hospital, Shanghai, China; 2 School of Medical Technology, Shanghai University of Medicine and Health Sciences, Shanghai, China; 3 Shanghai Pudong Hospital Affiliated to Fudan University, Shanghai, China; 4 Shanghai University of Medicine and Health Sciences, Shanghai, China; 5 Tongji University Affiliated Eastern Hospital, Shanghai, China

**Keywords:** mutation, colorectal cancer, health, low-abundance

## Abstract

Detecting low-abundance mutations is very important for cancer diagnosis and treatment. Here we describe an improved targeted sequencing analysis that dramatically increases sequencing depth. Seven colorectal cancer (CRC) patients and seven healthy adults were enrolled in this study. We examined genetic mutations in tissue samples from the central and peripheral regions of tumors from the CRC patients and in blood cells from the healthy adults. We observed that each CRC carried larger numbers of mutations more than previously estimated. These included numerous deletion mutations in the tumor tissue. While the cellular morphology in the surrounding normal colonic tissues was healthy, these cells also carried many mutations. Similarly, the blood cells from the healthy donors carried numerous mutations. These findings shed new light on the processes of tumorigenesis and aging, and also present a potentially effective method for detecting low-abundance mutations for cancer diagnosis and targeted treatments.

## INTRODUCTION

Cancers result in part from the accumulation of genetic mutations, which are caused by multiple intrinsic factors, such as errors during cell proliferation and DNA repair, and extrinsic factors, such as UV light and aflatoxins [1–3]. For example, a guanine-to-cytosine transversion at amino acid 12 of *KRAS* is specifically associated with human neoplasms [4]. Similarly, mutation of *RB*, the first successfully cloned tumor suppressor gene, triggers the occurrence of retinoblastoma [5]. However, the apparent and direct causal associations between genetic mutations and cancer are infrequent; in fact, the roles of most mutations in cancer remain obscure. Although there are useful online mutation databases, such as COSMIC, it is very difficult to account for all the mutations that occur in all genes in cancer [6]. Furthermore, there may be large numbers of mutations within each gene. For instance, there are hundreds of mutations within *TP53* [7]. Additionally, compared to wild-type genes, mutant genes may acquire new functions, most of which have not been clearly identified [8, 9].

It has been hypothesized that tumorigenesis arises when normal stem cells mutate and transform into cancer stem cells [10]. After years of continuous proliferation, mutations may occur that are selected when they confer a fitness advantage for cellular adaptation and evolution [11, 12]. Ultimately, the stem cells become cancerous when they acquire critical malignant features. Although this hypothesis explains the heterogeneity of cancerous tissues, the precise steps in this process have not been fully illuminated.

Mutation detection is playing a more and more important role in the diagnosis, prevention and treatment of cancer. For example, mutations in *KRAS* and *BRAF* are effective indicators for colorectal cancer (CRC) diagnosis and prognosis prediction [13]. Moreover, larotrectinib, a highly selective inhibitor that is widely used to treat cancer patients with mutations in Neuro Trophin Receptor Kinase (NTRK), was approved by the U.S. FDA last year [14]. Recently, a variety of effective methods have emerged for clinical mutation detection, including next-generation sequencing, third generation single-molecule sequencing, the amplification refractory mutation system (ARMS), and digital PCR [15–17]. FoundationOne CDx, for example, is a FDA-approved mutation detection kit based on targeted, massively parallel sequencing, which has been widely used in the diagnosis and treatment of cancer [18].

CRC is one of the most common and deadly cancers worldwide. Its incidence rate was ranked third in the United States in 2019 [19]. Due to the strong proliferative capacity and exposure to a complex environment, mutation rates are very high in CRC [1]. To investigate mutations in CRC, we have developed a modified method for targeted sequencing. By designing special primers, we have dramatically improved sequencing depth. In this report, we describe detection of mutant genes in cancer tissues from seven CRC patients and in blood cells from healthy donors and make comparisons between the two groups.

## RESULTS

### Clinical detection of seven CRC patients

**Figure 1:**
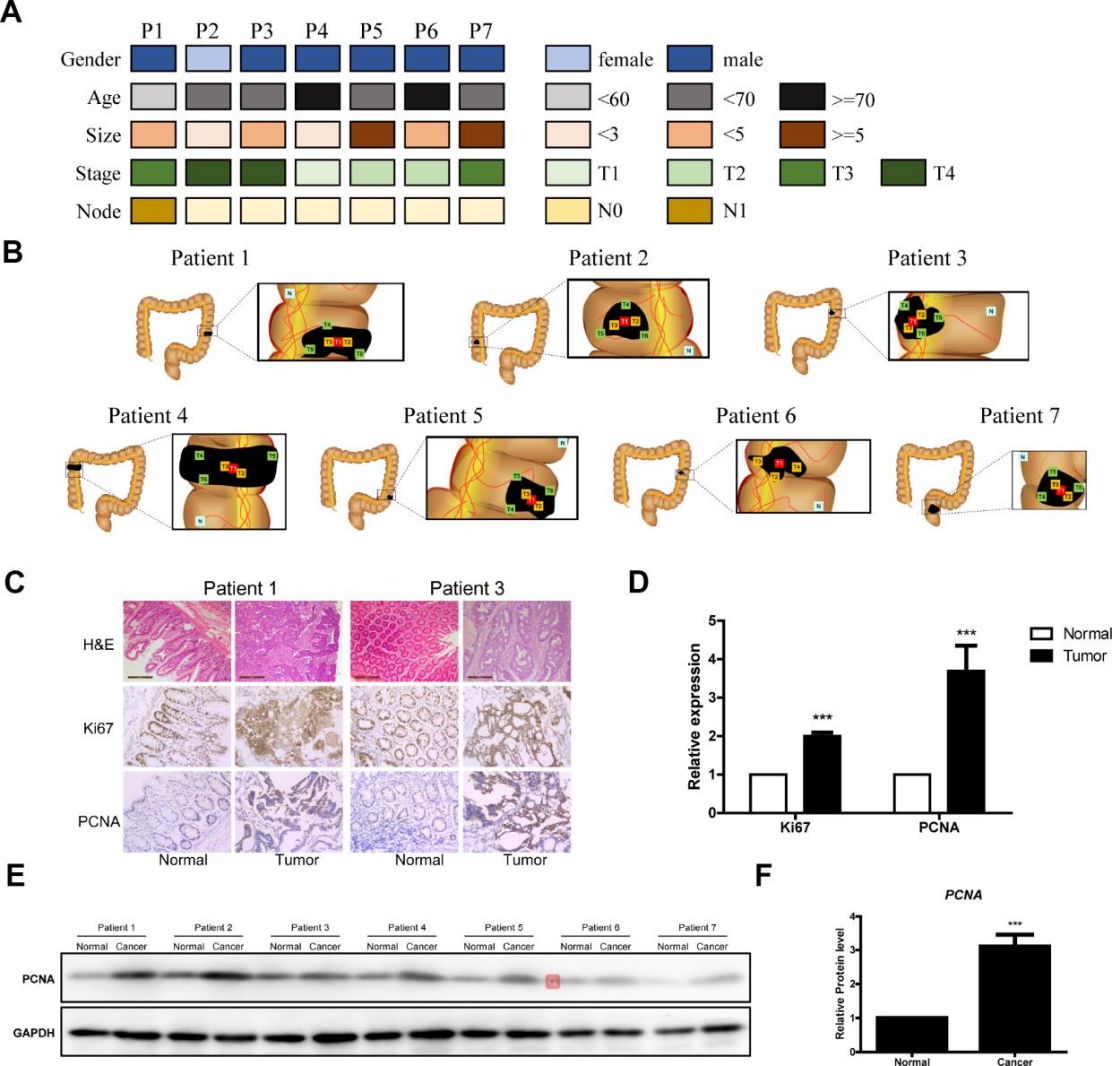
**Clinical data of the seven CRC patients.** (**A**) Clinical analyses of seven CRC patients. (**B**) Schematic diagram of the locations of the tumor tissues from the seven patients. (**C**) Representative images of H&E Staining, Ki67 immunostaining and PCNA immunostaining of samples from the seven patients. (**D**) Data analysis of Ki67 immunostaining and PCNA immunostaining of tumor tissue sections. (**E** and **F**) Western blot of PCNA in tumor tissue sections.

To explore the tumor mutation burden (TMB) of CRC among all cancers, we analyzed TMB in The Cancer Genome Atlas (TCGA) dataset. CRC is associated with one of the highest TMBs in TCGA (Supplementary Figure 1). We also enrolled seven patients with CRC and collected their clinical data (Figure 1A, Supplementary Table 1). For tumor sample preparation, we divided the tissue into several pieces (Figure 1B). The results of hematoxylin and eosin staining showed that the cell boundaries within the tumor tissues were not clear. By contrast, cells within normal tissues maintained good cellular morphology (Figure 1C). Finally, tumor tissues were deeply immunostained for Ki67 and PCNA, which are indicative of the proliferative capacity of the cells and suggests strong cell division (Figure 1C and 1D). The PCNA protein was sharply upregulated in tumor tissues as well (Figure 1E and 1F).

### Frequency of mutated genes in the seven CRC patients

**Figure 2:**
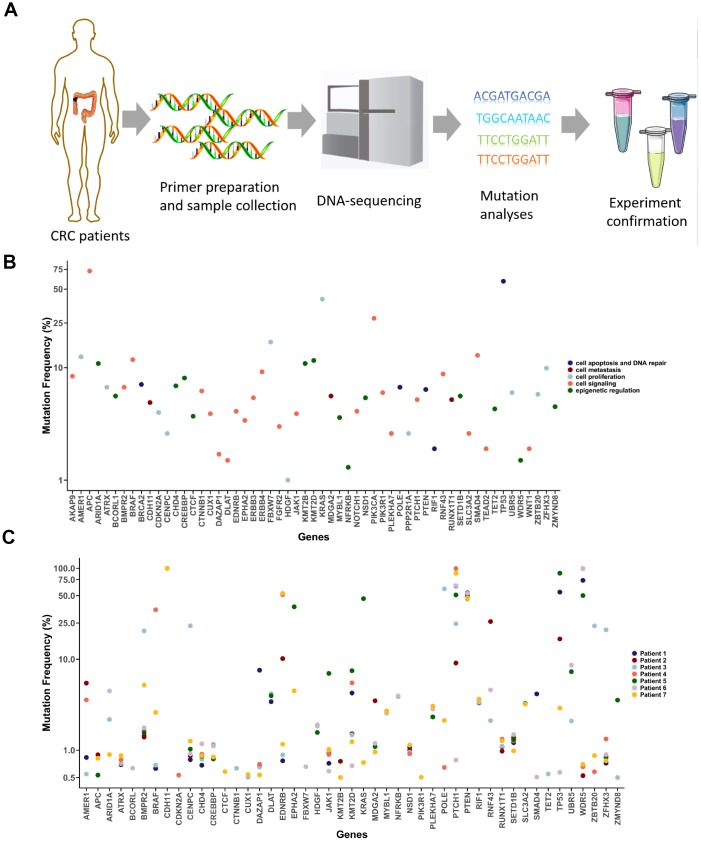
**Detection of cancer-associated mutations in T1 cancer tissues.** (**A**) Schematic diagram of the mutation detection method. (**B**) Selected CRC-associated genes. Mutation frequency data were acquired from the CRC project of TCGA. (**C**) Mutation detection in T1 tumor tissues from the seven CRC patients.

To detect low-abundance mutations in CRC, we used high throughput sequencing with collected DNA samples (Figure 2A). We selected the targeted DNA sequences based on the mutation frequency and the gene’s function (Figure 2B). The selected genes with a high mutation frequency and mutation hotspots were classified into five groups based to their function (Figure 2B). Because tumor sizes in CRC patients differ greatly, we used mutation data from T1 tumor tissue for further study. Interestingly, although we detected only 57 genes in total (27,976 total bases), 47 of them were mutated at least once in all patient samples, and the mutation probability in the tumors was very high (Figure 2C and Supplementary Figure 2). More than 20 mutant genes were detected in each patient sample. Moreover, the functions of the mutant genes varied widely in each patient (Supplementary Figure 2). Interestingly, the mutation frequency for several genes, including *CDH11* and *PTEN*, was quite high (Figure 2C). Because this may reflect Asian-specific genotypes rather than the mutations per se, we focused on genes with mutation frequencies <20%.

### Mutation analyses of the selected genes in different samples from the seven CRC patients

**Figure 3:**
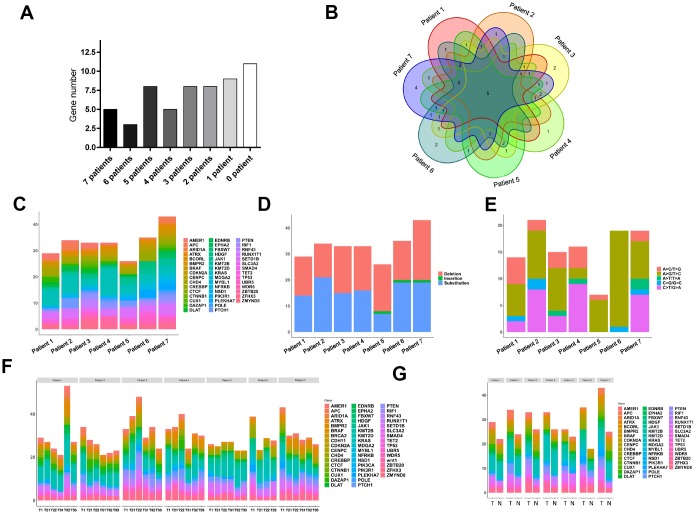
**Widespread cancer-associated mutations in tumor and normal tissues.** (**A**) Distribution of mutations in genes we selected in the seven CRC patients; the grayscale reflects the frequency of mutations. (**B**) Wayne diagram of the distribution of mutated genes in patients. (**C**) Distribution of mutated genes in the seven CRC patients. (**D**) Distribution of deletion and substitution mutations in the seven patients. (**E**) Distribution of subdivided substitutions in the seven patients. (**F**) Distributions of mutant genes in tissue samples from the central peripheral regions of the tumors in the seven CRC patients. (**G**) Distributions of mutant genes in samples from T1 tumors and normal colonic tissue from the seven CRC patients.

Among the 57 genes tested, 5 were mutated in all seven patients, while mutations in 10 genes were not detected in the selected sequences at all (Figure 3A). Unique mutated genes were those detected in only one patient. The Venn diagram in Figure 3B shows that there the most unique mutated genes were detected in patient 7, while patient 7 carried the largest number of mutations as well (Figure 3C). We found that the frequency of deletion mutations was high in these tumor samples, especially in patient 5, whose deletion mutations exceeded 50% of all the mutations (Figure 3D). Analysis of the substitution mutations showed that A>G substitutions were the most common, followed by A>C and then C>T (Figure 3E). Comparison of the genomic changes in the central and peripheral areas of the T1 tumor samples revealed no significant differences in the mutant genes between the central and peripheral parts of the tumor samples (Figure 3F). We then compared mutant genes between matched normal and tumor tissues from the CRC patients. Intriguingly, although the morphologies of normal and tumor tissues markedly differed (Figure 1C), the differences in their mutated genes were not significant (Figure 3G).

### Mutation detection in blood cells from seven healthy donors

**Figure 4:**
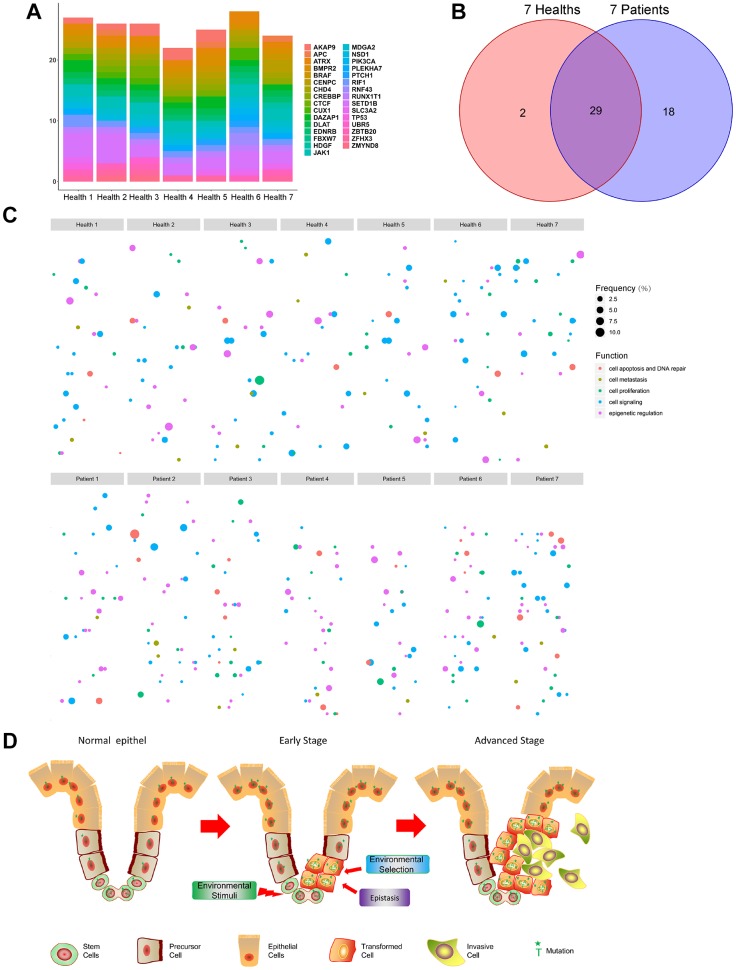
**Detecting mutations in normal blood cells from healthy donors.** (**A**) Distribution of selected mutant genes in the seven healthy donors. (**B**) Wayne diagram of the distribution of mutated genes in CRC patients and healthy donors. (**C**) Scatter plot of the mutated genes in CRC patients and healthy donors. (**D**) Schematic diagram. Stem cells in tissues proliferate over years and accumulate mutations. The majority of cells (including stem cells) carry mutant genes (Left). DNA replication and repair and environmental factors (UV, radiation, etc.) lead to the transformation of mutant stem cells into cancer stem cells, while environmental selection and epistasis influence mutations in the tumor (Middle). After continuous iterative selection, cancer stem cells eventually develop into malignant tumors (Right).

Because numerous mutations were found in the normal tissues from CRC patients, we speculated that similar mutations may occur in other normal tissues, such as blood. We therefore enrolled seven matched healthy donors and collected their blood cells. Our subsequent analysis revealed many mutations in these normal blood cells, with an average of about 20 mutant genes in each sample (Figure 4A). A large majority (93.5%) of the mutated genes in healthy donors were also detected in tumor tissues, suggesting that these mutations may be attributable to similar mechanisms, such as replication errors (Figure 4B). When we compared the mutated genes from the healthy donors and patients based on mutation frequency and gene function, we found that there was a large number of mutations in the healthy donors that were shared by CRC patients (Figure 4C). These results indicate that there may be many mutations in normal somatic cells in healthy people.

## DISCUSSION

Cancerous tumors are heterogeneous tissues that contain a variety of cell types, including normal stem cells, supporting cells, immune cells, endothelial cells and diverse tumor cells with different mutations [20, 21]. Consequently, we need to be able to detect low-abundance mutations within tumor tissues, as some tumor cells are in low abundance at the early stages of cancer [21]. Detecting these low-abundance mutations is very important for cancer diagnosis and targeted treatment. Targeted sequencing has been an effective method for detecting low-abundance mutations in cancer studies and in clinical applications [22]. We improved upon this method and increased the depth of sequencing by at least 10-fold, greatly enhancing the detection sensitivity for low-abundance mutations. For example, *EGFR*, which is usually mutated within exons 19 and 21 is the gene targeted by Gefitinib in non-small cell lung cancer. [23, 24]. However, after 5 months of treatment, 50% of patients will carry the T790M (ACG>ATG) mutation and need further Osimertinib treatment [25]. We propose that these patients carrying the high-abundance *EGFR* mutation in exons 19 and 21, may also carry a low-abundance T790M EGFR mutation. With the method described herein, the low-abundance EGFR T790M mutation can be detected earlier. It would therefore be of interest to test this speculation through further experimentation and clinical exploration.

Mutation accumulation is thought to be one of the most important factors contributing to tumorigenesis [26]. While the results of numerous studies are consistent with that idea, the precise steps in the process remain unclear [27, 28]. It has been reported that cancers derive from a single cancer stem cell through mutation [29]. These cancer stem cells then continuously proliferate, and the mutations accumulate [30]. Over several years, tumor cells acquire new functions, such as anti-apoptosis, increased migration, and immune resistance [31]. Interestingly, we found that there were no marked differences in the mutations between normal tissue and CRC tissue. That is, normal tissues contained more mutations than we expected, which is consistent with the idea that that mutations accumulate in human adult stem cells throughout life [1, 32]. Based on our findings and those of others, we speculate that in the sea of mutations, some cells suddenly obtain a decisive change, such as uncontrolled proliferation, due to environmental stimuli or an intrinsic mutation. The resultant malignant cells then begin to proliferate and quickly evolve (Figure 4D).

Aging is an eternal theme in medicine and health. Immune cells, secreted communication factors, and the shortening of telomeres partially explain this phenomenon [33, 34]. In addition, somatic tissues such as the normal human esophagus accumulate mutant clones with age [1]. Here we revealed that there are large numbers of mutations in the blood cells of healthy donors. Our present findings, together with earlier work [1], suggest that the accumulation of mutations in somatic cells with age is another important factor in aging, and it will be very interesting to identify the mechanisms underlying these mutations. Perturbation of DNA duplication and repair could cause accumulation of mutations [35, 36]. For instance, DNA lesions such as guanine N7 alkylation alter guanine hydrogen-bonding patterns in duplex DNA and inhibit DNA duplication [37]. These studies and our work partially explain the relationships among aging, mutations and tumors. However, the associations between mutations and other chronic diseases associated with aging, such as diabetes, cardiovascular disease, and neurodegenerative diseases, deserve further study.

Organ transplantation or regeneration is the ultimate treatment for many incurable diseases, such as liver, kidney, and heart failure [38, 39]. Induced pluripotent stem cell technology is thought to be a promising method for organ regeneration [40, 41]. However, unpredictable and uncontrollable carcinogenesis is a key problem with this method [42]. One possible reason is that the mixed cells with mosaic mutations may contain precancerous cells that can become malignant when exposed to the four iPS factors (Oct4, Sox2, Klf4 and c-Myc). It is therefore essential to detect mutations before iPS induction. Moreover, because the number of somatic cells carrying mutations increases with age, it may be necessary to store stem cells at a young age.

There are several limitations to this study. Though we have found the numbers of low-abundance mutations in CRC patients, the patient number is small, especially for various patients with different stages, different sexes and different age. Large-scale clinical research should be conducted to reveal more functions of low-abundance mutations in CRC. Besides clinical indicators, the backgrounds of the patients, such as ethnicity and eating habit, have important roles in the development of colorectal CRC [43]. It is of interest to study the roles of these background in low-abundance mutations. The tumorigenesis of CRC is a long-time period and illustrating the function of low-abundance mutations during the period is very important. Due to CRC’s slow development and the capacity for early diagnosis, tissue samples such as colorectal polyps and precancerous lesions could be collected during different stages of the disease [44]. Thus, it is very valuable to unveil a hidden world of low-abundance mutation and CRC tumorigenesis through large-scale sequencing.

In summary, we used a new targeted sequencing method to detect mutations in CRC tissues and matched normal colonic tissues. This approach also enabled us to reveal that there are numerous mutations in blood cells from healthy donors. These findings not only shed new light on the mechanism of tumorigenesis, but also revealed the universality of somatic cell mutation with aging. This method has a very high sensitivity for low-abundance mutations and may have potential applications for the diagnosis and treatment of tumors.

## MATERIALS AND METHODS

### Patients and samples

For this study, seven CRC patients and seven healthy adults at Zhoupu Hospital (Shanghai, China) were enrolled between July 2017 and July 2018. All samples were obtained with written informed consent at study entry. All methods used in this study were approved by the Research Medical Ethics Committee of Zhoupu Hospital. The tumor tissues and the matched normal tissues were collected as shown in Figure 1B. The freshly collected tissue samples were placed in cryogenic storage tubes (Thermo Fisher Scientific) and immediately stored in liquid nitrogen (Thermo Fisher Scientific). Blood samples from healthy adults were collected into EDTA anticoagulant tubes and then placed in cryogenic storage tubes in liquid nitrogen as well. Genomic DNA was extracted from frozen tumor tissues using a standard protocol (FG304, Shanghai Finegene Biotech, Shanghai, China). The quantity and quality of the DNA was assessed using NanoDrop 2000 (Thermo Fisher Scientific).

### Primer design for targeted sequence

To detect target regions, the reference regions of 57 genes are necessary for PCR [45]. The sequences of all these regions were downloaded from the UCSC Genome Browser (hg38; http://genome.ucsc.edu/). We designed the specific blunt hairpin primers of multiplex PCR as described previously [46, 47]. These primers contain two parts (Supplementary Figure 3). The first is the 3’-end containing about 20 bp, which constitutes the targeted region and has a melting temperature of 60-65°C (Supplementary Table 2). The second part is a universal sequence (18 bp), which enables the PCR products to be amplified by adapter primers to construct the sequencing libraries. The sizes of PCR product are around 200 bp. We evaluated the specificity of these primers as previously described [48].

### Adapter primers design

To construct the sequencing libraries with the different samples, we designed unique adapter primers containing Ion Torrent primer. These primers contained ten-base index sequences and universal sequences. All primers were synthesized by Biowing Applied (Shanghai, China). The adapter primers were HPLC purified and were supplied at standard desalting grade by Biowing Applied.

### Multiplex PCR

To amplify the 200-bp target region, the PCR mixture (40 μl) contained 1× reaction buffer (HotStart DNA polymerase (1.2 U), 2 mM MgCl_2_, 200 μM each dNTP and 2× KAPA2G Robust HotStart ReadyMix (Kapa Biosystems, USA), 1 μM each primer (), and 50 ng of genomic DNA. The PCR cycling protocol entailed: 95°C for 3 min and 20 cycles of 94°C for 15 s and 60°C for 4 min.

### Library construction

For the library construction, the PCR mixture (10 μl) contained: 1× reaction buffer (2 mM MgCl_2_, 0.4 U of KAPA2G Robust HotStart DNA polymerase), 200 μM each dNTP and 2× KAPA2G Robust HotStart ReadyMix (Kapa Biosystems, USA), 1 μM adapter primer, and 2 μl of template, which yielded the first round PCR products. The PCR cycling protocol entailed: 95°C for 3 min; 10 cycles of 94°C for 20 s, 65°C for 2 min, 72°C for 30 s; and 72°C for 5 min. The PCR products were purified using a TIANgel Midi Purification Kit (TIANGEN BIOTECH, Beijing, China).

### Ion torrent PGM sequencing

The sequencing was performed by Biowing Applied. Briefly, the purified PCR products were sequenced on a PGM platform using commercially available protocols and were then processed on a OneTouch 2 instrument. After enrichment using a OneTouch 2 ES station, the products were sequenced using a 318 chip and an Ion PGMTM Sequencing 200 Kit v2 [47].

### Sequencing data analysis

We separated all the sequencing reads according to their index combination information using a FASTX-Toolkit with the parameter that mismatch bases were less than 1 bp. We then trimmed out the index and adapter sequences using cutadapt software to generate target sequences for each sample. Thereafter, sequencing reads were mapped against the reference genome (hg38; http://genome.ucsc.edu/). For quality control, the filtration cutoff used a Phred-type quality score of Q20 (QPhred = 20) and the amplicons with > 5,000 sequencing depths were excluded in order to avoid extreme read depths [49, 50]. The detail of the sequencing such as the total number of reads were shown in Supplementary Table 3.

### H&E Staining

Hematoxylin and eosin (H&E) staining was performed to evaluate the histopathological structure of CRC tumor tissue. The samples were fixed in formaldehyde (10%), embedded in paraffin, cut into 4-μm-thick sections, stained with hematoxylin solution for 5 min. This was following by 5 dips in acid ethanol (1% HCl in 70% ethanol). After rinsing the sections in distilled water, they were stained with eosin solution for 5 min, dehydrated through a graded alcohol series, clearing in xylene, and finally mounted in neutral gum. The samples were observed under a Nikon fluorescence microscope (Tokyo, Japan).

### Immunohistochemical Staining

For immunohistochemistry, paraffin-embedded 4-μm sections first kept at 60°C for 24 h, deparaffinized with xylene and hydrating through an ethanol gradient (100%-70%), and incubated for 30 min in an antigen retrieval solution (Hangzhou Huabio Biotechnology Company; Hangzhou, China) and 3% H_2_O_2_. After being rinsed with water, the sections were incubated with the primary antibody (anti-Ki67 (1:50 dilution; ab197234, Abcam, Cambridge, UK) overnight at 4°C. They were then rinsed and incubated for 30 min with the secondary antibody (Hangzhou Huabio Biotechnology Company; Hangzhou, China) followed by 3,3′-diaminobenzidine (DAB) and hematoxylin. The immunostained sections were observed under a Nikon fluorescence microscope (Tokyo, Japan).

### Western blot

The protein of PCNA in the normal and tumor tissues were analyzed by Western blot. Briefly, the tissues were treated with RIPA buffer (high) (Solarbio, Beijing, China) in ice for 10min and were lysed with pestle in ice for 5 min. Total lysates were centrifuged and the protein concentration of the supernatant was tested with a BCA protein assay kit (Beyondtime, Nantong, China). 20 μg of total proteins were separated by SDS-PAGE and electro-blotted onto a PVDF membrane (Merck KGaA). The membranes were blocked with 3% BSA for 1 h at room temperature, and incubated with different primary antibodies: anti-PCNA (#ab18197; Abcam, Cambridge, UK) and anti-GAPDH (#ab181602; Abcam). After incubated with the appropriate secondary antibodies for 1 h at room temperature, the bands were visualized using an ECL assay kit (Amersham Pharmacia Biosciences) and observed with an LAS3000® Luminescent image analyzer.

## Supplementary Material

Supplementary Figures

Supplementary Tables

Supplementary Table 2
