# Supplementary material for: Low-abundance mutations in colorectal cancer patients and healthy adults
Source: Aging (Albany NY). 2020 Jan 12;12(1):808–24. doi: 10.18632/aging.102657 (PMC6977685; doi:10.18632/aging.102657)
Supplement: Supplementary Figures [file aging-12-102657-s003..pdf]

SUPPLEMENTARY FIGURES

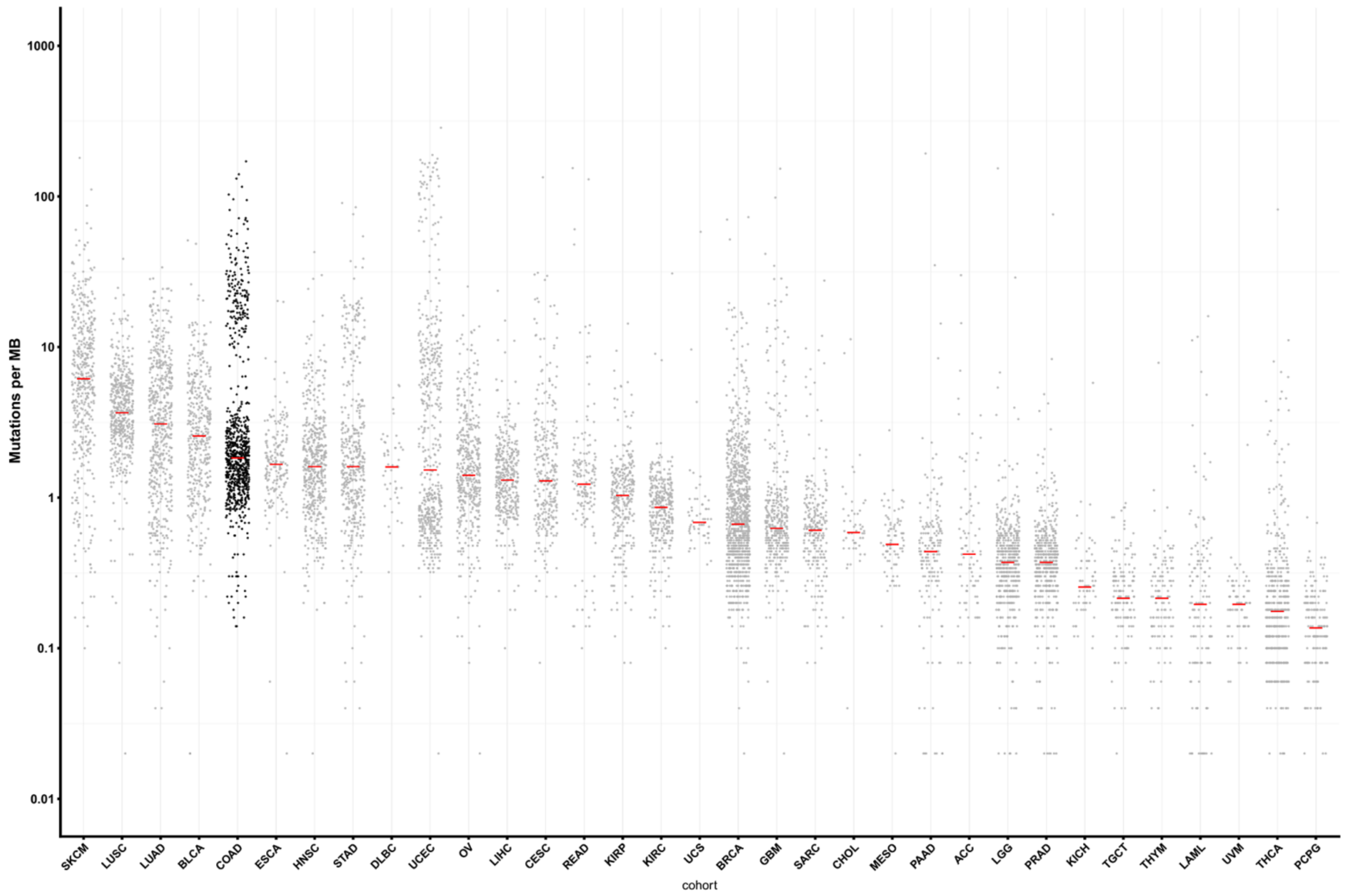

Supplemental Figure 1. The nonsynonymous tumor mutation burden across 33 cancer types in TCGA. Each dot corresponds to a tumor sample. The log10 scaled vertical axis demonstrates the number of nonsynonymous mutations per megabase. The 33 cancer types are ordered on the horizontal axis according to their median numbers of nonsynonymous mutations per megabase, which were represented by the short red horizontal lines. The COAD lied in the fifth column.

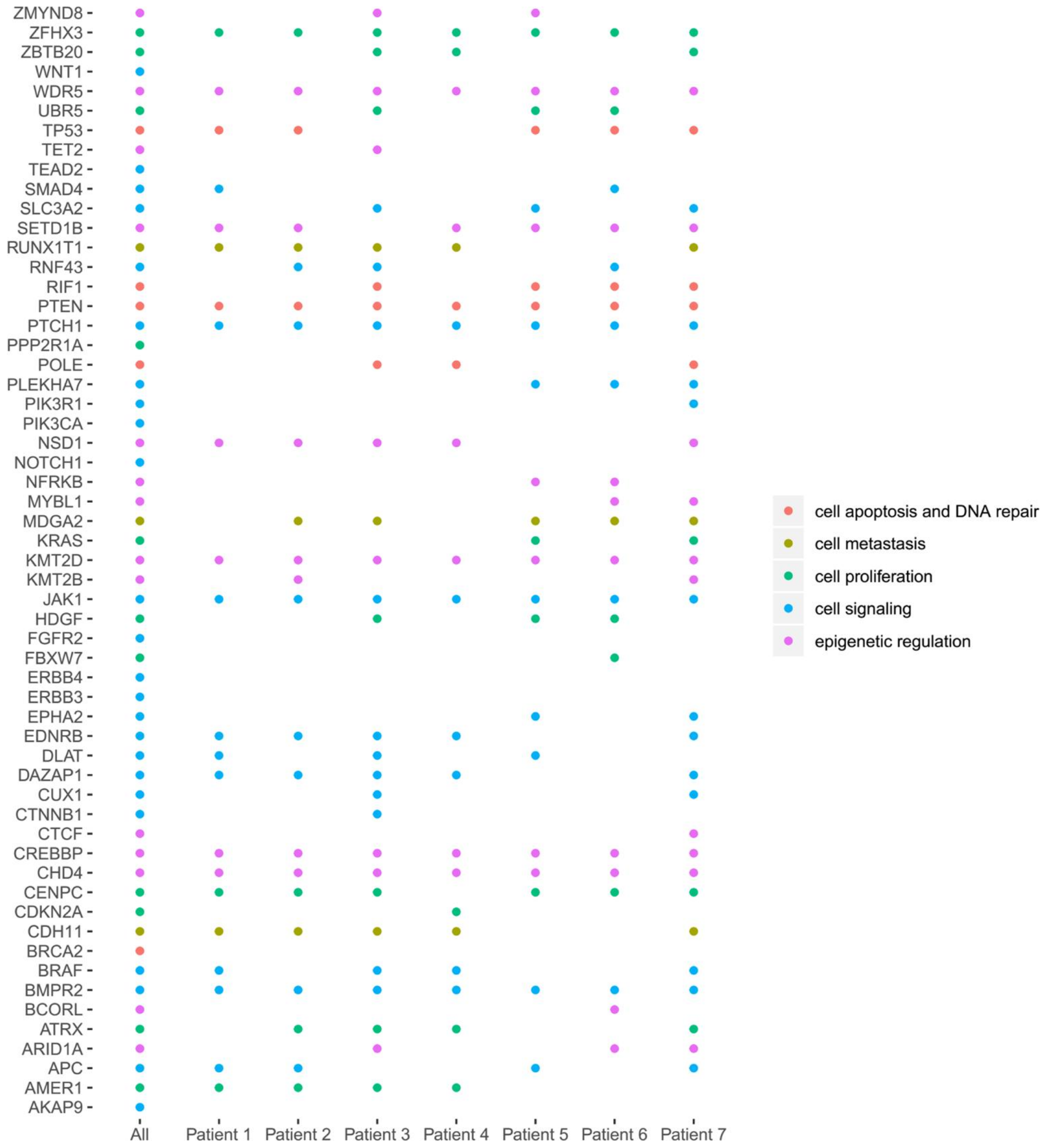

Supplemental Figure 2. Mutation detection of T1 tumor tissues from the six COAD patients. All the selected genes lie in the first column. The mutant genes that detected in each patients lied in the second to seventh column. All the detected mutant genes lied in the eighth column.

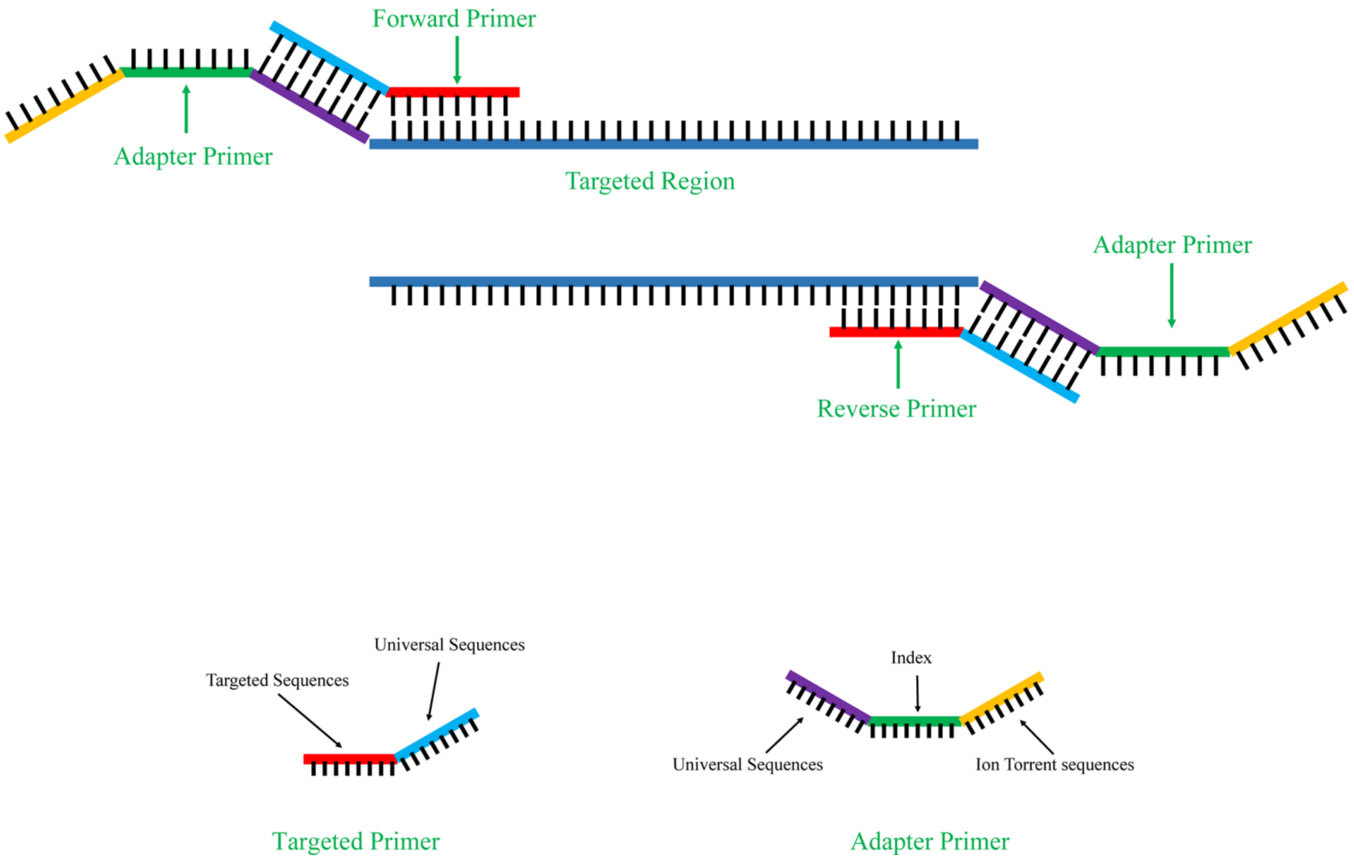

Supplemental Figure 3. Schematic diagram of primer design. Two primers were design in this study: the targeted primer and the adapter primer, as shown in methods section. The sequence of the targeted primer were shown in Supplementary Table.
