# Supplementary material for: Low-abundance mutations in colorectal cancer patients and healthy adults
Source: Aging (Albany NY). 2020 Jan 12;12(1):808–24. doi: 10.18632/aging.102657 (PMC6977685; doi:10.18632/aging.102657)
Supplement: Supplementary Tables [file aging-12-102657-s002..pdf]

SUPPLEMENTARY TABLES

Supplementary Table 1.

No.	Sex	Age	Tumor size	Stage	Lymph.Nodes.Positive	CT detection
201702011	female	65	1.5*1.5*0.5cm	III	1	1
201705053	male	52	4.5*4.5*0.5cm	IV	0	1
201701065	male	66	4.5*4.5*1.0cm	IV	0	1
201802060	male	73	1.5*1.0*0.5cm	I	0	1
201804011	male	62	5.0*4.0*3.0cm	II	0	1
201806006	male	81	4.0*1.0*0.8cm	II	0	1
201902005	male	64	6.5*3.5*1.5cm	III	0	1

Please browse Full Text version to see the data of Supplementary Table 2

Supplementary Table 2.

Supplementary Table 3.

Patients	sample	raw reads	raw data (bp)	Q30(%)	clean reads	clean data (bp)
Health 1	blood	2874958	431243700	98.60%	2834786	425217900
Health 2	blood	3555754	533363100	98.65%	3507699	526154850
Health 3	blood	3791302	568695300	98.55%	3736490	560473500
Health 4	blood	3465252	519787800	98.60%	3416689	512503350
Health 5	blood	2733798	410069700	98.66%	2697123	404568450
Health 6	blood	2374414	356162100	98.35%	2335284	350292600
Health 7	blood	2597040	389556000	98.51%	2558332	383749800
Patient 1	T1	8983730	1347559500	94.44%	8484669	1272700350
Patient 1	T21	3889436	583415400	94.61%	3679665	551949750
Patient 1	T22	6487360	973104000	95.23%	6177609	926641350
Patient 1	T31	5021792	753268800	95.15%	4778485	716772750
Patient 1	T32	5293576	794036400	95.10%	5034356	755153400
Patient 1	T33	7208058	1081208700	95.47%	6881547	1032232050
Patient 1	normal	10611972	1591795800	95.14%	10096302	1514445300
Patient 2	T1	12907630	1936144500	94.81%	12237581	1835637150
Patient 2	T21	643660	96549000	95.38%	613951	92092650
Patient 2	T22	6291702	943755300	94.59%	5951473	892720950
Patient 2	T31	5938210	890731500	95.01%	5641969	846295350
Patient 2	T32	12856498	1928474700	94.88%	12198651	1829797650
Patient 2	T33	11851226	1777683900	95.20%	11282304	1692345600
Patient 2	normal	26942700	4041405000	94.34%	25418950	3812842500
Patient 3	T1	6077548	911632200	96.13%	5842055	876308250
Patient 3	T21	9745696	1461854400	95.74%	9330659	1399598850
Patient 3	T22	4530982	679647300	95.81%	4341041	651156150
Patient 3	T31	4008834	601325100	94.92%	3805217	570782550
Patient 3	T32	6339228	950884200	95.03%	6024258	903638700

Patient 3	T33	13664850	2049727500	95.46%	13044423	1956663450
Patient 3	normal	7876790	1181518500	94.97%	7480867	1122130050
Patient 4	T1	37350466	5602569900	94.61%	35337186	5300577900
Patient 4	T21	30142032	4521304800	94.49%	28480857	4272128550
Patient 4	T22	12475154	1871273100	95.75%	11945368	1791805200
Patient 4	T31	15092174	2263826100	95.48%	14409462	2161419300
Patient 4	T32	10925402	1638810300	95.76%	10462200	1569330000
Patient 4	T33	10587636	1588145400	95.67%	10128681	1519302150
Patient 4	normal	29675188	4451278200	95.16%	28238376	4235756400
Patient 5	T1	2200670	330100500	95.30%	2097344	314601600
Patient 5	T21	5394972	809245800	95.81%	5169181	775377150
Patient 5	T22	2657258	398588700	95.28%	2531863	379779450
Patient 5	T31	3108924	466338600	95.41%	2966341	444951150
Patient 5	T32	7477154	1121573100	95.33%	7127832	1069174800
Patient 5	T33	4484220	672633000	95.21%	4269267	640390050
Patient 5	normal	2867686	430152900	95.51%	2739052	410857800
Patient 6	T1	1228996	184349400	95.62%	1175189	176278350
Patient 6	T21	4565322	684798300	95.42%	4356374	653456100
Patient 6	T22	2607780	391167000	95.78%	2497784	374667600
Patient 6	T23	1288430	193264500	95.64%	1232260	184839000
Patient 6	normal	1706826	256023900	95.97%	1637963	245694450
Patient 7	T1	6903882	1035582300	95.88%	6619402	992910300
Patient 7	T21	12933792	1940068800	95.92%	12405758	1860863700
Patient 7	T22	7628024	1144203600	96.39%	7352945	1102941750
Patient 7	T31	12530866	1879629900	95.76%	12000062	1800009300
Patient 7	T32	3809072	571360800	95.70%	3645240	546786000
Patient 7	T33	8493836	1274075400	95.63%	8122311	1218346650
